# Three Years with the Army of the Potomac

**Published:** 1911-06

**Authors:** 


### "Three Years with the Army of the Potomac"

DURING the time which the late William Warren Potter spent as a surgeon with the army of the Potomac in the civil war he wrote a series of letters which later he made into a manuscript volume of reminiscences under the general title, "Three Years with the Army of the Potomac:" A personal military history.

It was Dr. Potter's desire that during his life this manuscript be used solely for the entertainment of his friends and those who were close to him were permitted to read its pages replete with interesting experiences and thrilling with the activities of war.

Dealing wholly with the life of a surgeon the writing is of special interest to the profession and it will now be published in serial form in the JOURNAL beginning with the July issue. It will be published as written by Dr. Potter in the long past days.

---

DR. CARLOS MACDONALD and Dr. Austin Flint, experts appointed by the commission engaged in examining into the conduct of state prisons and reformatories, have reported their findings of an examination of the inmates of the Mattawan State Hospital. They examined all but thirty-two of the seven hundred and ninety-three inmates and have stated in official report that "in no instance did we find an inmate wrongfully detained or subjected to inhuman or improper treatment at the hands of officers or attendants." Complaint had been made that many of the inmates of the institution were improperly detained. This was the natural outcome of the many silly reports emanating from the Thaw family's efforts to free that interesting inmate of Mattawan, Harry Thaw, who shot and killed Stanford White. Thaw was one of those who was not examined by the alienists having refused to appear.

There was recommendation made that the institution be placed on a purely hospital basis.

WALTER E. DURYEA, of Glen Cove, L. I., who lived twelve years with a broken neck and whose case attracted the attention of the medical profession of the world died at Montclair, N. J., in May. He left property amounting to considerably over one million dollars. Aside from a few personal bequests to members of his immediate family the estate goes to hospitals and Miss Eleanor Peregrin who was his nurse during the time of his disability.

She received $50,000 outright, a trust fund of $30,000 and two houses. She is then made residuary legatee the latter provision bringing to her relatively $1,000,000. Hospital bequests are: Nassau Hospital Association, Mineola, L. I., $25,000; Roosevelt Hospital, New York, $15,000; a trust fund of $75,000 devised by Mr. Duryea's father is given to the Nassau Hospital; Mountain Side Hospital, Montclair, N. J., $7,000; Montclair Fresh Air Convalescent Home, $2,000.

